# Parliament and the Profession

**Published:** 1915-11-06

**Authors:** 


### PARLIAMENT AND THE PROFESSION.

### No Badges for Doctors' Chauffeurs.

On October 26 Mr. Tyson Wilson asked whether chauffeurs of medical men who were attending military hospitals two or three days a week would be allowed to wear badges. Mr. Lloyd George stated in reply that it was not considered that the conditions governing the use of war badges had been satisfied in these cases.

### Insurance Act Economies.

The Chairman of the Joint Committee of Insurance Commissioners stated in the House of Commons on October 26 that a number of administrative changes, which it is hoped will result in substantial savings, has been carried out in the department since the outbreak of war. Proposals for further economy have been submitted to the Committee on Public Retrenchment, but the majority of these proposals can only be effected by legislation.

### Medical Students and Lord Derby's Appeal.

"The view of the War Office is that fourth and fifth year medical students should continue their studies, but that students in the first, second, and third years must consider for themselves what answer they should make to the recruiting appeal addressed to them, and not regard themselves, so far as the War Office is concerned, as under the duty of continuing their medical studies." This statement was made by Mr. Tennant in the House of Commons on November 2, in reply to questions by Mr. Snowden and Sir Philip Magnus. It is in harmony with previous pronouncements.

### Mental Hospitals for Soldiers.

The Under-Secretary of State for War informed Mr. Touche on October 26 that the administration of mental hospitals in which soldiers are being treated is under the full control of the War Office. Mr. Tennant also stated that the arrangements now in force adequately safeguarded the interests of uncertifiable soldiers, and he added that the cases of mentally injured soldiers admitted to such institutions were treated as nerve-shock cases in which recovery was possible or probable. He also assured the House that the number of certifiable cases relatively to the size of the Army was very small.

### Head Wounds in French Hospitals.

It was suggested by Mr. Bryce, in a question asked in the House of Commons on October 28, that, while the percentage of head wounds in French hospitals had fallen to *nil*, the percentage of such wounds in British hospitals is still very great, amounting in some hospitals to 15 per cent. The inference to be drawn from these facts—if they are facts—is that the steel helmets which are provided for French soldiers serve their purpose very efficiently. The Under-Secretary of State for War informed Mr. Bryce that he was obtaining information as to the percentage of such wounds in British hospitals, and he hoped it would soon be available. It would be a mistake, however, to suppose that the wearer of a steel helmet is immune from head wounds by modern weapons.

### The Martyr-Nurse.

Touching the murder of Miss Cavell, a question was asked in the House of Commons on October 27 by Mr. Ronald McNeill, who desired to know whether it was intended to take any steps to convey to the Military Governor of Brussels that, when opportunity offered, he would be held personally responsible by His Majesty's Government for "the quasi-judicial assassination of Miss Cavell." In his reply Lord Robert Cecil reminded hon. members of the Prime Minister's assurance that due reparation would be exacted from all persons, whatever their position, who could be shown to have maltreated our prisoners in Germany. "That pledge," added the Under-Secretary of State for Foreign Affairs, "still holds good, and applies with twofold force in the case of the savage murder of a noble woman."

### The Payment of Panel Doctors' Fees.

The vexed question of the delay in the settlement of National Health Insurance practitioners' accounts has again been raised in the House of Commons. On October 27 Sir Philip Magnus asked Mr. Charles Roberts, as administrative head of the Insurance Act, whether he could see his way to accelerate the payment of the balance due to panel doctors for services rendered in the year 1914. Mr. Roberts, in his reply, said he feared he could only assure panel doctors that the Commissioners were using their best endeavours to expedite the settlement by all means within their power, but he could not bind himself down to a date even approximately. While we can quite understand that there are difficulties in the way of calculating the precise sums due to practitioners, it appears to be a most unbusinesslike procedure on the part of a public authority to keep their creditors waiting more than ten months while they find out to what extent they are indebted to them. The mischief is the doctors have to meet their liabilities even more promptly than in normal times.

### Grotesque Misrepresentations.

From time to time fantastic statements are published in the lay Press concerning the danger of inoculation. For instance, it was recently reported that at a public meeting in Manchester Mr. H. G. Chancellor, M.P. (Haggerston), said it was "well known that pneumonia often supervened on [anti-typhoid] inoculation. In some cases inoculation brought on madness; in others it had led to invalidity, men were totally unfitted for their duties, and were cast diseased upon the world, incapable of gaining their livelihood, and without a penny of reward for the suffering they had undergone." Presumably with this fanatical utterance fresh in his memory, Mr. Partington in the House of Commons on October 28 asked the Under-Secretary of State for War the definite question "whether there had been reported to the War Office any, and, if so, how many, cases of the kind referred to by Mr. Chancellor." Mr. Tennant's reply, which we quote in full, was as follows: "Such reports as those mentioned in the question have been made to the War Office by the hon. member for the Haggerston Division, but not, so far as I am aware, by any other person. Investigations have been made, and it has been proved that the individuals spoken of as having died as a result of inoculation against enteric fever have in fact died of some quite ordinary disease. In particular my attention has been called to a speech delivered on October 16 by the hon. member mentioned, and I should like to say with reference to the statements then made, some of which are reproduced in the question, that they are not only without foundation, but are grotesque misrepresentations of the result of a treatment from which the Army has derived incalculable benefits." It is, we suppose, too much to hope that even this categorical denial will check the activities of wrong-headed fanatics.

